# Edward Wentworth Luther

**Published:** 1911-04-08

**Authors:** 


The death was announced on March 28, at Saint Jean de Luz, of Edward Wentworth Luther, Deputy Inspector-General of Hospitals, R.N., who had retired. The son of an Irishman, he was educated at Dublin, and took his diploma at the Royal Irish Colleges in 1872.

